# Safety and effect of Neuroform Atlas stent in the treatment of symptomatic intracranial stenosis: A single-center experience

**DOI:** 10.1016/j.heliyon.2021.e08040

**Published:** 2021-09-21

**Authors:** Orazio Buonomo, Enricomaria Mormina, Antonio Armando Caragliano, Agostino Tessitore, Antonio Pitrone, Mariano Velo, Marco Cavallaro, Carmela Visalli, Francesca Granata, Carmela Vadalà, Sergio Lucio Vinci

**Affiliations:** Department of Biomedical Sciences and Morphological and Functional Images, University of Messina, Messina, Italy

**Keywords:** Intracranial stenosis, Neuroform Atlas stent, Acute ischemic stroke, Transient ischemic attack, Atherosclerosis

## Abstract

**Background:**

Vascular intracranial stenosis (IS) is a significant cause of acute ischemic stroke (AIS). This single-center study aims to show that symptomatic IS treatment by using the Neuroform Atlas stent (Stryker neurovascular, Kalamazoo, MI, USA) could be effective in reducing vessel stenosis.

**Methods:**

Ten patients affected by AIS or TIA, in the vascular territory of high-grade intracranial atherosclerotic lesions (>70% of vessel stenosis), older than 18-year-old, were treated by implanting a Neuroform Atlas stent (diameter of 4.5mm in 80% and 4mm in 20%). 70% of the patients underwent pre-stenting intracranial angioplasty.

**Results:**

Patients were between 54.8 and 83 years old (mean 68.46y ± 8.44y), 70% males and 30% females. At admission, 50% of all patients had an AIS and 50% a TIA. Restoration of the stenotic lumen was obtained after the endovascular procedure. The percentage mean of vascular stenosis was 83.7% ± 6.09% before treatment (t0), 52.2% ± 10.42% at the end of treatment (t1) and 46.2% ± 8.28% at the follow-up (t2). The IS percentage mean reduction between t0 and t1 was 31.5% ± 7.31%, and between t1 and t2 was 6% ± 5.47%, t0 and t2 of 37.5% ± 7.38%. Percentage reduction of IS was highly significant between time t0 and t1 (*p* = 0.005), and t0 and t2 (*p* = 0.005), also with a significant reduction between t1 and t2 (*p* = 0.012). No patient had experienced an increase of the ischemic area in the vascular territory of the target vessel at 3 months from the initial assessment. 10% of patients experienced a 3-months negative outcome (mRS = 5), 90% experienced a favorable outcome (mRS ≤2).

**Conclusions:**

Intracranial stenosis endovascular treatment with Neuroform Atlas stent provides encouraging results, with a statistically significant association between the vascular caliber improvement and the endovascular treatment.

## 1 Introduction

In Western Europe, from 1990 to 2010 a significant reduction of incidence, mortality, disability-adjusted life year (DALY), and the mortality-to-incidence ratio of ischemic stroke occurred. Specifically, most recent data demonstrates an age-standardized incidence of acute ischemic stroke in a range between 97.1 - 127.3 per 100000 people/year and a mortality-to-incidence ratio in a range between 0.26-0.29 [1]. At the same time, intracranial vascular stenosis has become a significant cause of acute ischemic stroke and, recently, the prevalence of this condition is 31% in an elderly population with common cardiovascular risk factors, with a percentage of vascular stenosis higher than 50% in 9% of cases. Indeed, by dividing the percentage prevalence by the severity of intracranial stenosis (IS), Suri et al. described 4.2% of patients had stenosis between 50% and 69%, 3.1% between 70 and 99%, and 1.7% a total occlusion. This study showed that there was a statistically significant association between IS and several risk factors, such as advanced age, black race, elevated systolic blood pressure, and elevated low-density lipoprotein (LDL) levels. Conversely, high hyperdensity lipoprotein (HDL) and the use of cholesterol-lowering drugs were found to be protective factors of IS, while no correlation was found on smoking and body mass index (BMI) [2].

Patients with IS due to atheromatous plaque are at high risk of recurrent ischemic events. Despite carrying out appropriate medical therapy, 38.2% of patients experienced a new cerebrovascular event within two years: 13.7% of ischemic stroke and 24.5% of transient ischemic attack (TIA). These percentages increase in patients with IS causing a hemodynamic deficit [3].

In our single-center study, we aim to show in ten patients affected by symptomatic IS the safety effect of using the Neuroform Atlas stent (Stryker neurovascular, Kalamazoo, MI, USA) with and without association with angioplasty with the NeuroSpeed double-lumen Percutaneous Transluminal Angioplasty (PTA) balloon (Acandis, Pforzheim, Germany).

## 2 Material and methods

The study received ethical approval from the Institutional Review Board (Interhospital Ethical Committee of Messina – A.O.U. “G. Martino” Policlinico Messina). The primary endpoints were technical success and procedure success. The technical success was defined as good functioning of the devices, with a reduction of the degree of stenosis immediately after the procedure, allowing a valid distal flow with a good parenchimography of the downstream territory. The procedure success was defined as technical success without intraprocedural stroke or death or any type of complications.

Secondary endpoints were considered late complications such as symptomatic restenosis, modification of intracranial stent, and neurological worsening at follow-up.

### 2.1 Clinical and diagnostic criteria for patients selection and pre-treatment IS evaluation

From February 2019 to June 2020, ten Caucasian patients admitted to the Stroke Unit of our institute were selected. Before the Endovascular Treatment (EVT), informed consent was obtained. If the patient was unable to give explicit consent, the accompanying relatives were in any case made aware of the procedure.

In our study, we selected only patients with acute ischemic stroke or TIA, in the vascular territory of high-grade intracranial atherosclerotic lesions (more than 70% of vessel stenosis), both of anterior or posterior circulation, with an age >18 years old and without upper age limit.

We excluded from the study patients with other IS than the treated one, patients who developed collateral neoanastomotic vessels, an Alberta stroke program early CT score evaluated with MRI (ASPECT-DWI) < 6 (for anterior circulation AIS), a posterior circulation ASPECT (pcASPECT) (for posterior circulation AIS) < 6 without brainstem bilateral involvement, with an AIS onset >24h.

At admission, each patient was studied with non-contrast CT and Magnetic Resonance Imaging (MRI) (DWI-EPI b0/b1000 s/mm^2^; FLAIR-2D; TOF-3D). CT angiography (CTA) was performed subsequently to assess the patency of the intra- and extracranial vessels. The percentage of stenosis of the target lesion was described firstly by CTA and, afterward, by Digital Subtraction Angiography (DSA).

Clinical status at follow-up was assessed by experienced neurologists and neuroradiologists, who jointly evaluated clinical and imaging data.

The clinical and laboratory evaluation included anamnesis, routine blood tests, physical exam, neurological exam with assessment of the values of the major stroke scales, ASPECT-DWI or pcASPECT at admission, National Institutes of Health Stroke Scale (NIHSS) pre- and post-AIS/TIA, and modified Rankin Scale (mRS) pre- and post-AIS/TIA.

The clinical and demographic data of each patient are summarized in Table 1.

**Table 1:** Clinical, neuroradiological and pre/post-treatment data.

	1	2	3	4	5	6	7	8	9	10	Mean ± SD or %
Age	77,5	63,25	72,58	83	60	65	67,08	54,83	67,25	74,08	68,46 ± 8,44
Sex	F	M	M	M	F	F	M	M	M	M	70% Male
Smoke	No	Yes	Yes	No	Yes	No	Yes	Yes	Yes	Yes	70% Yes
Dyslipidemia	Yes	No	Yes	No	No	Yes	No	Yes	Yes	Yes	60% Yes
Diabetes	No	No	No	Yes	No	Yes	No	No	No	No	20% Yes
Hypertension	Yes	Yes	Yes	Yes	Yes	Yes	Yes	Yes	Yes	Yes	100% Yes
Target Vessel	BA	ICA	BA	ICA	BA	BA	VA	ICA	BA	BA	60%BA, 30%ICA, 10%VA
IS length mm	15	2	2	16	6	2	3	10	3	15	7,4 ± 6
IS t0 (%)	83	92	79	76	84	83	91	75	91	83	83,7 ± 6,09
IS t1 (%)	61	68	49	40	52	41	66	41	47	57	52,2 ± 10,42
IS t2 (%)	52	51	49	40	43	31	61	40	45	50	46,2 ± 8,28
IS t0-t1 (↓%)	22	24	30	36	32	42	25	34	44	26	31,5 ± 7,31
IS t1-t2 (↓%)	9	17	0	0	9	10	5	1	2	7	6 ± 5,47
IS t0-t2 (↓%)	31	41	30	36	41	52	30	35	46	33	37,5 ± 7,38
AIS vs TIA	AIS	TIA	AIS	AIS	AIS	TIA	AIS	TIA	TIA	TIA	50% AIS
NIHSS-pre	2	0	30	9	16	0	30	0	0	0	8,7 ± 12,4
NIHSS-post	1	0	30	1	4	0	14	0	0	0	5 ± 9,79
ASPECT-DWI	-	10	-	10	-	-	-	10	-	-	10
pcASPECT	9	-	6	-	8	10	8	-	10	10	8,71 ± 1,49
mRS-pre	1	0	1	1	1	1	0	2	0	1	0,8 ± 0,6
mRS-post	1	0	5	1	2	1	2	2	0	1	1,5 ± 1,4
Atlas size mm	4,5 × 30	4 × 30	4,5 × 21	4,5 × 30	4,5 × 30	4,5 × 21	4 × 24	4,5 × 21	4,5 × 21	4,5 × 30	80% 4,5mm Ø
PTA	Yes	Yes	No	No	No	Yes	Yes	Yes	Yes	Yes	70% Yes
IPMT	Tb + i	No	F500 Tb + i	Tb + i	F500 Tb + i	Tb + i	F500 Tb + i	No	No	No	60% IPMT
PPMT	DAPT A40	DAPT	DAPT	DAPT A40	DAPT A40	DAPT A40	DAPT A40	DAPT† A80	DAPT	DAPT A80	100% PPMT

SD = Standard Deviation; BA = Basilar Artery; VA = Vertebral Artery; ICA = Intracranial Internal Carotid Artery; IS = intracranial stenosis; t0,t1,t2 indicate different time points: before procedure (t0), immediately after procedure (t1), 3 months after procedure (t2); ↓% = Percentage reduction; AIS = Acute Ischemic Stroke; TIA = Transient Ischemic Attack; NIHSS = National Institute of Health Stroke Scale; -pre: before procedure; -post: post procedure; ASPECT-DWI = Alberta ​stroke program early CT score evaluated with MRI; pcASPECT = posterior circulation ASPECT; mRS = modified Rankin Scale; PTA = Percutaneous Transluminal Angioplasty; IPMT = Intraprocedural Medical Therapy; PPMT = Postprocedural Medical Therapy; DAPT = Dual AntiPlatelet Therapy (clopidogrel 75mg/die + Acetylsalicylic Acid 100mg/die); DAPT† = Dual AntiPlatelet Therapy (ticlopidine 250mg x2/die + Acetylsalicylic Acid 100mg/die); A40 = Atorvastatin 40mg/die; A80 = Atorvastatin 80mg/die; F500 = flectadol 500mg e.v; Tb + i = tirofiban bolus and 12h infusion (bolus of 10 μg/kg administered over 3 min followed by a 12h infusion of 0.15 μg/kg/min); Ø = diameter.

### 2.2 Endovascular procedure

All patients underwent EVT under general anesthesia, with strict control of the average blood pressure values. During the procedure, naïve patients for antithrombotic therapy were given 500 mg of Flectadol (ASA) endovenous and tirofiban bolus and 12h infusion (bolus of 10 μg/kg administered over 3 min followed by an infusion of 0.15 μg/kg/min) based on weight's patient according to the cardiologic intermediate protocol [4].

Tirofiban bolus and infusion were administered to patients who were already on therapy with ASA 100 mg/day. No intraprocedural drug was administered to patients who were already on DAPT. These data are shown in Table 1.

All endovascular procedures were performed in our single-center institution under general anesthesia and by two senior interventional neuroradiologists together, with more than 10 years of experience in intracranial stent deployment. No patient underwent thrombolysis and no thrombectomy attempt was performed before treating the underlying stenosis.

An 8 Fr. right femoral access was obtained in all patients with a short introducer using the Seldinger technique. All patients have been studied with a complete cerebral angiography with a 5 Fr. diagnostic catheter before the procedure. The procedures were all performed in triaxial technique using a 6F long sheath (Neuron Max 088 Penumbra, USA) and a Navien 6 Fr. 0.072 inches 105-cm ID distal intracranial catheter (Covidien Vascular Therapies). Hemostasis of the puncture site was obtained with the Angio-Seal device (St. Jude Medical) in all patients.

### 2.3 Materials

Neuroform Atlas stent (Stryker Neurovascular, Kalamazoo, MI, USA) is a low profile open-cell stent with a hybrid design; this device can be delivered through a 0.0165″ or 0.017” catheter.

The Neuroform Atlas Stent was not designed for the treatment of intracranial vascular stenoses but has some technical characteristics that make it suitable for this purpose, although an off-label treatment.

The structure of the Neuroform Atlas is an alternation of crowns made up of 8 and 12 struts. The 8-struts crown has a thicker wire to ensure an optimal vessel-wall apposition. The 12-struts crown has a thinner thread, which is responsible for optimized stent conformability. The alternation of open and closed cell structures makes the Atlas a hybrid design stent.

The Neuroform Atlas is a Nitinol-based stent, not visible under fluoroscopy and it has 3 proximal and distal radio-opaque markers.

During deployment, the stent maintains the length that is detectable inside the microcatheter, almost without foreshortening, thus providing high landing precision [5].

It is suitable for vessels with diameters between 2 and 4.5 mm. The available stent diameters range from 3 to 4.5 mm with stent lengths of 15, 21, 24, and 30 mm [6].

In our cohort of 10 patients, we implanted 4.5 mm (diameter) Neuroform Atlas in 8 patients and 4 mm (diameter) Neuroform Atlas in 2 patients as shown in Table 1.

The intracranial stent was released by NeuroSpeed ® PTA balloon catheter (Acandis), except for the 30% of patients who did not perform angioplasty; in this latter case, Neuroform Atlas was deployed through a 0.0165″ or 0.017″ microcatheter (Excelsior SL-10 or XT-17, Stryker neurovascular, Kalamazoo, MI, USA) over a 0.014” neurovascular micro guidewire.

The NeuroSpeed catheter is an over-the-wire double-lumen PTA balloon in the range of 1.5–4 mm. The balloon has a central lumen of 0.0165 inches, the useable length of 150 cm, balloon length of 8 mm, is semi-compliant, and allows PTA at the nominal size with standard pressure and diameter change more or less 0.3 mm based on the inflation pressure [7].

The selection of the angioplasty device size was based on the native diameter of the target vessel and the length of the stenotic lesion.

### 2.4 Postprocedural evaluation and medical therapy

After PTA and subsequent stent placement, each patient was assessed by digital subtraction angiography. Each patient underwent intensive surveillance in the Stroke Unit until discharge and received the best medical therapy according to comorbidities. All patients were treated with DAPT (90% with ASA 100 mg/day + clopidogrel 75 mg/day and 10% with ASA 100 mg/day + ticlopidine 250 mg x 2/day). 70% of pts were treated with oral lipid-lowering therapy (5/10 with atorvastatin 40 mg/day and 2/10 with atorvastatin 80 mg/day). The post-procedural medical therapy of each patient is summarized in Table 1.

### 2.5 Radiologic and clinical follow-up

After the endovascular procedure, all patients were assessed with a neurological examination (including estimation of NIHSS and mRS).

We evaluated IS percentage before and immediately after the procedure, using DSA; in our paper, these two time points will be respectively indicated as “t0” and “t1”.

All patients underwent neuroradiological follow-up, with CTA, 3 months after the endovascular procedure; in our study, this latter time point will be indicated as “t2”.

We evaluated 100% of the lumen considering the diameter of the target artery assessed immediately before the stenotic tract. Three experienced neuroradiologists evaluated the percentage of IS at each time point; the IS measurement average for each time point was chosen.

### 2.6 Statistical methods

Descriptive statistics as the arithmetic mean, median, range (minimum and maximum for continuous variables and *n* and percentage for discrete variables), graphs, and patient listings were used to evaluate and summarize data.

Since the number of statistical observations was small (10 patients) non-parametric tests were used, regardless of the type of variable. Consequently, non-parametric approaches were used.

To evaluate percentage changes of IS between different time points about specific patients characteristics (smoke exposure, diabetes, dyslipidemia) Kruskal-Wallis test and Logistic regression model were used. Wilcoxon's signed-rank test was used to evaluate whether the endovascular treatment with Neuroform Atlas was significant in determining a percentage reduction of IS between different time points (t0 vs t1; t0 vs t2; t1 vs t2). A value of *p* < 0.05 was considered statistically significant.

## 3 Results

Ten patients were selected for the study. Patients were between 54,8 and 83 years old (mean 68,46y ± 8,44y), 70% males and 30% females. Due to high-grade intracranial atherosclerotic lesions (>70% of vessel stenosis) in the anterior or posterior circulation (3/10 intracranial internal carotid artery; 6/10 basilar artery; 1/10 intracranial vertebral artery), 50% of all patients had an acute ischemic stroke and the other 50% had TIA.

All patients suffered from arterial hypertension and none had coagulation or genetic disorders. 70% of patients were already on anti-thrombotic therapy at the time of enrollment (3/10 in Acetylsalicylic Acid [ASA] 100 mg/day and 4/10 in dual antiplatelet therapy [DAPT-ASA 100 mg/day + clopidogrel 75 mg/day]). 3 out of 10 pt at the time of the cerebrovascular event were already in therapy with statins (2/10 pts - 40 mg/day and 1/10 pt - 80 mg/day).

IS length means was 7.4 mm ± 6 mm, with a range of 2 and 16 mm and a median of 4.5 mm.

We obtained a good improvement of the caliber of the target vessel with a restoration of the stenotic lumen compared to pre-procedural conditions. The percentage mean of vascular stenosis was 83.7% ± 6.09% before treatment (t0), 52.2% ± 10.42% at the end of treatment (t1) and 46.2% ± 8.28% at the follow-up (t2). The IS percentage mean reduction between t0 and t1 was 31.5% ± 7.31%, and between t1 and t2 was 6% ± 5.47%, t0 and t2 of 37.5% ± 7.38%.

Wilcoxon's signed-rank test showed a highly significant (*p* < 0.01) percentage reduction of IS between time t0 and t1 (*p* = 0.005), and t0 and t2 (*p* = 0.005), also with a significant reduction (*p* < 0.05) between t1 and t2 (*p* = 0.012).

Kruskal-Wallis tests showed that in our population there were no significant differences in the percentage reduction of IS accounting for different patients characteristics: smokers and non-smokers (*p* = 0.566), diabetics and non-diabetics (*p* = 0.189), patients with and without dyslipidemia (*p* = 0.915), and sex (*p* = 0.359) and age differences (*p* = 0.437).

Logistic regressions showed that in our population smoke exposure (*p* = 0.139), diabetes (*p* = 0.172), dysplipidemia (*p* = 0.335), age (*p* = 0.127), and sex (*p* = 0.335), did not cause a different response in terms of the percentage reduction of IS.

In our study, pre-stenting intracranial angioplasty with Neurospeed was performed in 7 out of 10 patients (70%). Technical and procedure success, set as the primary endpoints of our study, were obtained in 100% of treated patients; all patients completed the radiological follow-up at 6 months.

There was no ischemic or hemorrhagic stroke related to the procedure nor intraprocedural deaths. No patient had experienced an increase of the ischemic area in the vascular territory of the target vessel at 3 months from the initial assessment. No patient was subjected to a subsequent endovascular treatment after the intracranial stenting procedure.

1 out of 10 patients experienced a 3-months negative outcome (mRS = 5), while 9/10pts experienced a favorable outcome (mRS ≤2).

## 4 Discussion

Intracranial atherosclerosis is one of the most frequent causes of stroke in the world, especially in the Asian population. Three main causes determine an ischemic stroke following an IS, distal hypoperfusion, arteriovenous embolism, and extension of atheromatous plaques to the host of perforating arteries [8].

Atherosclerosis localized to the middle cerebral artery was found to be the most frequent cause of stroke associated with IS. The proximal part of the intracranial internal carotid artery was the second artery most affected by the pathological process [9].

In the work of Kim et al. IS was also divided into various subgroups by analyzing possible differences in risk factors and stroke pathophysiology between intracranial vs extracranial stenosis of the anterior circle vs the posterior one [9].

In the same study, metabolic syndrome was found to be a risk factor associated with intracranial rather than extracranial stenosis, but only in posterior circulation strokes. On the other hand, strokes of anterior circulation were found to be predominantly associated with an arterio-arterial embolic disease while posterior circulation strokes were more frequently related to ostial occlusion of perforating branches following the extension of atheromatous plaques [9].

Recent literature has shown that large artery atherosclerosis is the second leading cause of recurrent stroke, secondary only to cardioembolism [10].

It has been found that CTA is the most accurate imaging method to carry out the diagnosis of IS, and specifically, it has high sensitivity and specificity in identifying stenosis > or = 50% of the vascular lumen [11].

In our single-center study, we report optimal technical success (100%) of endovascular treatment of intracranial symptomatic vascular stenosis, employing Neuroform atlas stent alone or in combination with Neurospeed balloon, as an off-label treatment.

For our work, we decided to use the Neuroform Atlas stent since it presents many features that make the endovascular treatment of IS possible with a good profile of safety and efficacy. This stent does not have a distal guide and can be released into a low-profile microcatheter (0.165 inches). These characteristics could be very useful in IS treatment, helping in a safe stent placement limiting the risk of iatrogenic damage, also considering that a clear visualization of the distal target vessel is not always possible (e.g. preocclusive stenosis). Its proximal and distal markers are radiopaque inside the microcatheter and allow one to perform a good deployment of the stent that remains open into the vessel without shortening or lengthening [5]. Despite the low number of patients presented in our study, it is worth notice that technical success was achieved in all patients with stability or an improvement of the NIHSS score and mRS score in most of the cases; only one patient presented a bad outcome (mRS = 5) having a relatively low pc-ASPECTs (pc-ASPECTs 6) with a discrete stroke already consolidated.

Moreover, the significance of IS percentage reduction of t0 vs t1 (p < 0.01) and t0 vs t2 (p < 0.01) suggests that this approach for IS treatment could be beneficial for patients both in acute and chronic phases. Neuroform Atlas hybrid cell stent configuration does not provide itself a high radial force, but oversizing the diameter of the stent could increase its radial force. For this reason, stents with higher nominal diameter and length values were chosen in our study to achieve the highest radial force for the stent type [12]. The Neuroform Atlas stent with its cell configuration is helpful in a proper apposition to the vascular walls and providing a low metal burden in the arterial space, makes intrastent thrombosis unlikely.

Further, we found an IS percentage reduction between t1 and t2 (p < 0,05), showing that the described technique could give a crucial contribution to the slight continuous improvement of IS percentage reduction over time.

Over the years many authors have tried to analyze the possible role of surgical/interventional treatments in IS: in the early 80's the extracranial to intracranial bypass trial, which compared the extra-intracranial bypass between the superficial temporal artery and the middle cerebral artery with the medical therapy performed with cardio aspirin in 1377 patients, was published. The neurosurgical procedure did not lower the stroke rate compared to medical therapy with aspirin as a global assessment and was also associated with a worse outcome than a single antiplatelet therapy in patients with middle cerebral artery stenosis [13].

Furthermore, as predictable, it has been scientifically evidenced that the presence of multiple ischemic lesions in the vascular territory of a stenotic intracranial artery was a risk factor associated with a greater recurrence of stroke [14].

WASID trial highlighted that antiplatelet drugs have fewer adverse effects when compared to warfarin in patients with IS. However, the high percentage of recurrent cerebrovascular events prompted to seek endovascular therapy that could reduce the recurrence of stroke [15].

Specifically, it has been shown that IS >70%, early onset of symptoms (<17 days) and female sex was statistically associated with the onset of ischemic stroke in the vascularization territory of a stenotic vessel [16].

Over the years, several research groups have evaluated the safety and efficacy of endovascular treatment of IS in comparison to the best medical therapy. The first study on this topic was the SSYLVIA one which showed that the use of NEUROLINK system was associated with a high endovascular success rate. The patients treated in this study experienced stroke in 6.6% at 30 days and in 7.3% between 30 days and 1 year after treatment. Nevertheless, in follow-up evaluation, a significant restenosis rate of the target vessel was found in 35 % of cases, even if 61% of these were asymptomatic [17].

Subsequently, the WINGSPAN study assessed the safety and efficacy of the endovascular treatment of IS with nominal pressure angioplasty performed with Gateway balloon, followed by the subsequent release of Wingspan stent. With an opposite trend to the SSYLVIA study over time, in this case there has been a reduction in the stenosis rate of the target vessel; in addition, fewer intra-procedural adverse events occurred. The patients treated in the WINGSPAN study experienced a stroke in the territory of vascularization of the target vessel at 6 months in 7.1% of cases compared to 14% found in patients treated with NEUROLINK system [18].

Consequently, multicenter randomized trials where both balloon-mounted stent (VISSIT) and the Wingspan system (SAMMPRIS) were assessed, leading to a stop of the growing enthusiasm generated in the context of vascular treatment of IS [19, 20].

Specifically, the VISSIT trial observed an important rate of periprocedural adverse events in the stent group compared with the group of patients on medical therapy (24.1% vs 9.4% at 30 days and 36.2% vs 15.1% at 1 year). In the specific case of this study, the operator's experience (not adequately assessed) begins to be evaluated as an important bias as well as the technical limitation rate related to the device used [19].

It has also been reported how interventional neuroradiologists' experience could also play a pivotal role in technical procedural success [21].

In our study, Neuroform Atlas stent was deployed only by experienced neuroradiologists, with more than 10 years of experience in intracranial stent deployment.

The SAMMPRIS multicenter trial is a cornerstone in the evaluation studies of the best treatment of symptomatic IS. This work revealed the superiority of best medical treatment over medical therapy in addition to endovascular treatment with Wingspan stent. After an average follow-up duration of 32.4 months, 23% of patients in the stenting group vs 15% of patients in the best medical therapy group had a new cerebrovascular event. Important attention towards the control of multiple cardiovascular risk factors with combined medical therapy could have contributed to these results. In particular, careful monitoring of LDL levels treated with rosuvastatin, careful control of blood pressure values treated with common antihypertensive drugs, and, above all, with an accurate double anti-aggregation medical therapy with aspirin and clopidogrel continued up to 90 days.

After the preliminary release of the SAMMPRIS results, the Food and Drug Administration has approved the use for humanitarian purposes of the Wingspan device for patients with IS between 70%-99%, who had experienced two or more cerebrovascular events despite aggressive medical therapy and where the last event had occurred more than 7 days before endovascular treatment [20].

To confirm what has been described, the final data of the WEAVE (a Wingspan post-market surveillance trial) have been recently released. These data have shown that with expert interventionists, a more accurate patient selection, and the on-label use of Wingspan stent, excellent results can be obtained with a periprocedural stroke, bleeding, and death rate (<72 h) of 2.6% (2 patients over 152) [22].

Two other studies have been recently published about the endovascular treatment of IS in a setting of AIS and specifically as a rescue treatment after failure of mechanical thrombectomies procedures and as an alternative treatment of IS [23, 24].

The described technique consisted of intracranial stenting with Neuroform atlas stent in coordination with the use of the Gateway angioplasty balloon (Boston Scientific, San Leandro, CA, USA). The described technique was very similar to the technique that we have described in this study and obtained very good results, despite the low number of patients involved.

As already emphasized by the authors of these papers the presented technique has the huge advantage to perform stenting and angioplasty of IS without a need of intracranial exchange maneuvers; this point could be very crucial in obtaining a procedure without complication and is determined by the use of an over the wire balloon as Gateway balloon or, as in our experience, the Neurospeed balloon catheter. The stent placement by using the Neurospeed double-lumen balloon, for IS endovascular treatment, represents a novelty of our study; only 7 out of 10 patients were treated with this technique.

## 5 Limitations

Our study has some major limitations. First, it is a single-center study with a small sample of patients, limiting the statistical power of our results. Secondly, the retrospective nature of the study did not allow to test for other hypotheses, or to evaluate IS at follow-up with DSA. Notably, the target vessel stenosis was assessed for each patient at follow-up with CTA, while a DSA was not available for this purpose. Measurements at follow-up were performed according to strict criteria, by using the same CTA acquisition protocol for each patient, and by reporting the mean value of three measures performed by three different experienced neuroradiologists. Nonetheless, we are aware that a slight difference in measurements, especially at follow-up (with a metallic stent placed in the target vessel), could have affected the statistical power of our study, and we address this issue as a drawback of the study.

## 6 Conclusion

In our single-center experience, we obtained encouraging clinical and angiographic results in endovascular treatment of IS due to atheromatous disease with the use of an intracranial stenting technique with Neuroform Atlas stent with or without Neurospeed PTA balloon catheter.The IS evaluation before treatment (t0), after treatment (t1), and at follow-up (t2) revealed a statistically significant association between the vascular caliber improvement and the endovascular treatment. More data are mandatory to confirm and evaluate the real clinical benefit of endovascular treatment of IS and specifically with the above-mentioned technique.

## Declarations

### Author contribution statement

Orazio Buonomo, Antonio Armando Caragliano: Conceived and designed the experiments; Performed the experiments; Analyzed and interpreted the data; Contributed reagents, materials, analysis tools or data; Wrote the paper.

Enricomaria Mormina: Conceived and designed the experiments; Analyzed and interpreted the data; Contributed reagents, materials, analysis tools or data; Wrote the paper.

Agostino Tessitore: Conceived and designed the analysis; Analyzed and interpreted the data; Contributed analysis tools or data; Wrote the paper.

Antonio Pitrone: Conceived and designed the experiments; Performed the experiments; Contributed reagents, materials, analysis tools or data.

Mariano Velo: Performed the experiments; Contributed reagents, materials, analysis tools or data.

Carmela Visalli: Analyzed and interpreted the data; Contributed reagents, materials, analysis tools or data; Wrote the paper.

Marco Cavallaro, Francesca Granata, Carmela Vadalà, Sergio Lucio Vinci: Analyzed and interpreted the data; Contributed reagents, materials, analysis tools or data.

### Funding statement

This research did not receive any specific grant from funding agencies in the public, commercial, or not-for-profit sectors.

### Data availability statement

Data included in article/supplementary material/referenced in article.

### Declaration of interests statement

The authors declare no conflict of interest.

### Additional information

No additional information is available for this paper.
