# Corrigendum to “Safety and effect of Neuroform Atlas stent in the treatment of symptomatic intracranial stenosis: A single-center experience” [Heliyon Volume 7, Issue 9, September 2021, Article e08040]

**DOI:** 10.1016/j.heliyon.2025.e43463

**Published:** 2025-06-04

**Authors:** Orazio Buonomo, Enricomaria Mormina, Antonio Armando Caragliano, Agostino Tessitore, Antonio Pitrone, Mariano Velo, Marco Cavallaro, Carmela Visalli, Francesca Granata, Carmela Vadalà, Sergio Lucio Vinci

**Affiliations:** Department of Biomedical Sciences and Morphological and Functional Images, University of Messina, Messina, Italy

In this article, the authors omitted a statement in their article regarding ethical approval number for the research presented in this publication, which is required under the policies of the journal. The authors confirm that ethical approval was obtained from Interhospital Ethical Committee of Messina – A.O.U. “G. Martino” Policlinico Messina for the research under the following reference number: V Prot. 77-21.

Original version of the Ethical Approval statement in section 2. Materials and methods:

*The study received ethical approval from the Institutional Review Board (Interhospital Ethical Committee of Messina – A.O.U. “G. Martino” Policlinico Messina)*.

To address this, the specific ethical approval reference number (V Prot. 77-21) has been added to the statement.

The correct version of the Ethical Approval statement should be as below.

The study received ethical approval from the Institutional Review Board (Interhospital Ethical Committee of Messina – A.O.U. “G. Martino” Policlinico Messina) *under reference number V Prot. 77-21*.

The authors apologize for the error.

## Declaration of competing interest

The authors declare no conflict of interest.

